# Prevalence and predictors of men's involvement in pregnancy care in Modakeke, Southwest Nigeria

**DOI:** 10.3389/fgwh.2024.1337094

**Published:** 2024-03-14

**Authors:** Aminat Omolara Akinyemi, Elhakim Adekunle Ibrahim

**Affiliations:** ^1^ Department of Demography and Social Statistics, Faculty of Social Sciences, Obafemi Awolowo University, Ile-Ife, Nigeria; ^2^ Department of Demography, College for Health, Community and Policy, The University of Texas at San Antonio, San Antonio, TX, United States

**Keywords:** pregnancy-related care, antenatal care, men’s involvement, male participation, male partner involvement (MPI), male perception, gender norms, Nigeria

## Abstract

**Introduction:**

Maternal mortality in developing countries remains a major public health concern and lack of men's support for their spouse during pregnancy contributes to this adverse outcome. This study examined the level and determinants of men's involvement in pregnancy care in Modakeke, Southwest Nigeria.

**Methods:**

A community-based cross-sectional, mixed-methods study involving quantitative and qualitative data. A multistage sampling strategy was used to select the study participants. The study involved 414 male respondents interviewed using a structured interviewer-administered questionnaire. The interview was complemented with one focus group discussion facilitated using an unstructured interview guide. Quantitative data were analyzed using descriptive and inferential analytical techniques while qualitative data were explored using thematic content analysis.

**Results:**

Findings revealed a 55% level of involvement in pregnancy care among the participants. However, involvement rates were higher among those who were younger, married, from monogamous family, with secondary or tertiary education, low-income earners, and holding positive perception about nontraditional gender roles. Multivariate logistic regression estimates indicated significantly lower odds of involvement among unmarried men than the married but increased odds among those who had secondary or higher education relative to the uneducated, and among those whose partners received antenatal care from multiple providers compared to from health facilities only. Furthermore, significantly reduced odds of involvement were associated with holding nonpositive perceptions towards accompanying spouse to antenatal care clinic and being involved in general pregnancy care as opposed to holding positive perception. Perceived challenges undermining male involvement as highlighted during focus group discussion include time constraints due to job demands, prohibitive sociocultural norms, rebuke and unconducive health facility environment.

**Conclusion:**

The level of involvement in pregnancy care is suboptimal among the study participants with considerable sociodemographic, socioeconomic and sociocultural dimensions. Enhancing men's involvement in pregnancy care requires community-based awareness-raising interventions that emphasize crosscutting benefits of male partner's participation in pregnancy-related care and address known sociocultural barriers impeding active involvement.

## Introduction

Maternal mortality rate remains unacceptably high in Nigeria, despite remarkable progress recorded in recent years. The latest report by the United Nations ranks Nigeria as the country with the highest maternal mortality rate in the world, with an estimated 814 maternal deaths per 100,000 live births as of 2020 (1). Besides, the country bears the greatest burden of maternal mortality globally, accounting for more than a quarter of all pregnancy-related deaths worldwide. Furthermore, a Nigerian woman faces 1 in 22 lifetime risk of dying from maternal causes as compared to 1 in 4,900 for their peers in the developed countries. Although most of these deaths are preventable through timely and adequate use of healthcare services during pregnancy outcomes (2), men's support during this period can enhance access to those essential interventions and improve perinatal outcomes (3–6). Research, for instance, established that accompanying a pregnant woman to antenatal care raised the odds of commencing antenatal visits during the first trimester, adequate use of antenatal care services, health facility delivery and presenting for postnatal check-ups (3). Thus, understanding the factors that influence men's engagement is of public health significance.

Despite its cross-cutting benefits, myriads of evidence from community-based studies done in Nigeria and elsewhere often indicate suboptimal level of men's participation in pregnancy-related care across the reproductive continuum (4, 7–10). For example, an investigation done in Agege, Southwest Nigeria reported 58.6% involvement rate in antenatal care among male partners (7). Likewise, evidence of 56.9% men's participation rate in pregnancy related care was documented in Ibadan, Southwest Nigeria (11, 12). Meanwhile, Erhabor and co-authors (8) found 27.2% engagement level in maternity care in Benin City, Southsouth Nigeria. Besides, studies from other countries have reported varying participation levels of 20% in Dodoma Region, Central Tanzania (9), 38.2% in the Bench Sheko zone, Southwest Ethiopia (10), 61.7% in Kashan city, Iran (13) and 70% *in Sekondi, Ghana* (14)*.* This portrays the need for enhanced men's role in reproductive health matters in many societies as advocated at the 1994 International Conference on Population and Development held in Cairo, Egypt (15).

Men's involvement in pregnancy care has been associated with several factors. Extant studies indicate that participation of male spouses is considerably influenced by age, family structure, type of marriage, occupation, ethnic and religious affiliations, educational attainment, level of income, perception and attitude towards involvement, and health care systems (3, 11, 16–19). For example, educational and economic disparities can play a critical role in shaping men's involvement in pregnancy care as men with higher levels of education and income may have more resources and ability to participate actively (7, 11). Also, men's own perceptions and expectations about their roles during pregnancy and the prevailing socio-cultural norms and stereotypes that delineate roles across gender divides can influence participation as many cultures typically feminize activities relating to pregnancy and childbirth (8, 12, 19, 20). Unhospitable attitude of maternity care staff, exclusion of men from obstetric care activities and unconducive facility environment may have negative impact on men's level of engagement (10, 14, 21).

Nevertheless, the dynamics of men's involvement in pregnancy care are yet to be explored in most social contexts in Nigeria despite their socioculturally entrenched role as the chief decision-maker in all family matters including those related to pregnancy and childbirth (8, 10–12, 16). Moreover, evidence on the role of several factors such as age, marriage type, religious and ethnic affiliations, and occupation on men's participation rate remains inconclusive (7, 10, 11). In addition, most investigations on male involvement have been conducted from women's perspectives (14, 16). This study aimed to contribute to the evolving body of knowledge being the first in the study area to assess the prevalence and predictors of men's involvement in the pregnancy care. Findings from this investigation will inform context-specific policies and interventions aimed at improving maternal and child health outcomes in the study area and other settings with similar socioeconomic and sociocultural configurations.

## Methods

### Study design, setting and population

This study employed a cross-sectional, community-based mixed-method research design involving quantitative and qualitative approaches of data collection. The primary study was conducted in a semi-urban town of Modakeke, the administrative headquarters of Ife-East Central Local Council Development Area, situated within the historic Ile Ife Kingdom in Osun State, Southwest, Nigeria. The primary inhabitants are Yorubas with fair representation of other ethnic groups. Rooted in Yoruba culture, the sociocultural fabric of the town delineates traditional gender roles which impedes male involvement in pregnancy care and considers prenatal care activities as exclusive preserve of women. However, societal norms elevate men as household heads and providers, tasked with meeting financial and essential needs, reflecting entrenched gender dynamics in the community (11, 12). Although most of the population is literate, the major economic activities are trading and farming. Modakeke has a projected population of 119,529 residents based on the 2016 national census figure of Nigeria as published by the World Population Review at https://worldpopulationreview.com/countries/cities/nigeria. Administratively, Modakeke is divided into 10 political wards. Men aged 20–59 years whose wife or partner had a live birth in the last 5 years and had lived in the study area for 6 months were the target population for the study.

### Sample size calculation and sampling strategies

The minimum sample size (n) of 335 respondents was derived using single population proportion formula [*n* = (z_α_^2^p(1–p))/e^2^] where z_α_ connotes the standard normal deviation corresponding to 95% confidence level and probability α of 1.96, *p* represents 32.1% prevalence of men's participation in pregnancy care, and e^2^ indicates 5% error margin (22). The 32.1% participation rate established by Iliyasu et al. (23) was adopted given paucity of evidence on the subject in the study area in line with previous studies done in similar social contexts (11, 12). However, 500 respondents were targeted to compensate for potential non-response, increase precision of estimates and ensure inclusion of diverse demographic constituents of the study location.

A multi-stage sampling technique was employed to enroll eligible respondents into the study. The first stage of the enrolment process involved random selection of one neighborhood from each of the ten political wards constituting the Development Area. Accordingly, each of the neighborhoods was allocated 50 respondents using the equal probability approach. In the second stage, simple random sampling was used to select the first housing or business unit to be visited. Using a random number generator, the fifth unit from the main entrance into the community was chosen and every tenth was subsequently visited. In units where there were multiple eligible and willing respondents, random selection by balloting was applied to enroll the respondent to be interviewed, while units where eligible participants were unavailable were replaced with the adjoining ones. Meanwhile, commitments were secured from 15 participants interviewed in five randomly selected neighborhoods for a follow-up group discussion.

### Ethical considerations

Ethical clearance was obtained from the Health Research Ethics Committee, Institute of Public Health, Obafemi Awolowo University, Ile-Ife, Nigeria (IPC/OAU/12/2328). Also, approval to conduct the study was granted by the Primary Healthcare Board of Ife-East Central Local Council Development Area, Modakeke, Osun State, Nigeria (OS/IECLCDA/PHC/01/2023). Respondents were enrolled after the objectives of the study had been explained and informed consent obtained. The participant's right of withdrawal from the study without any penalty was respected and the anonymity and confidentiality of the data generated were strictly maintained.

### Data collection and management

The quantitative data for this research was obtained using a structured questionnaire that was scripted, administered and stored via KoboToolbox mobile data collection platform. The questionnaire, delineated into 3 major sections, adapted validated contents and constructs relevant to the present investigation from the previous studies conducted in similar contexts (11, 12). The first section of the questionnaire elicited information on respondents’ demographic and socio-economic characteristics, the second section extracted responses on actual involvement in pregnancy care while inquiries relating to attitudes and perceptions about involvement in pregnancy care were addressed in the last section. The questionnaire items were examined for content validity through expert assessment, while content reliability was ascertained using Cronbach's alpha test (α = 0.71) and pilot testing of the instrument on a purposively selected sample of men. The questionnaire was administered by a team of 10 research assistants with university-level training in social research and previous experience in similar surveys. Data management activities were concurrently done using Stata software version 14.2 (24). A total of 471 out of 500 targeted respondents were successfully interviewed translating to 94% response rates. Meanwhile, the qualitative data were generated from a focus group discussion session facilitated by AOA using an open-ended, semi-structured interview guide. The interview guide comprised 2 sections which the first documented the basic demographics of the discussants while the second explored participants lived experiences in relation to involvement in pregnancy care, type of supports provided during pregnancy, factors affecting men's participation and societal attitudes and perceptions of partner's involvement in pregnancy-related care. This phase of the study involved 10 discussants out of 15 male partners who agreed to participate in the session during the quantitative data collection phase. The interview was held at a community civic center and lasted for about 60 min. The session was tape-recorded and augmented with field notes taken by AOA and a research assistant to document non-verbal cues and key concepts.

### Operational definition

The outcome variable for the study was men's involvement in pregnancy care measured dichotomously as “not involved” and “involved”, respectively coded as 0 and 1. A respondent was considered as having been involved in pregnancy care if he had accompanied his spouse to antenatal care clinic more than once and had provided a combination of financial, domestic, and/or emotional supports during the period of pregnancy (7, 8, 11, 23, 25, 26).

Meanwhile, a set of correlates that may be associated with involvement were identified based on the published literature (7, 8, 11, 23, 25, 26). The variables considered were classified into three categories as sociodemographic (i.e., age group, marital status, family type, religion, and ethnicity), socioeconomic (i.e., educational status, employment status, occupation group, income level and place of antenatal care), and sociocultural (i.e., perception about attendance of antenatal care clinic and perception about involvement in general pregnancy care).

### Analytical strategy

This study leveraged descriptive, inferential and thematic analyses. Descriptive statistics presented as frequencies and percentages were used to summarize the characteristics of the study participants. Pearson's Chi-square test and bivariate logistic regression were used to identify explanatory variables significantly associated with involvement in pregnancy care. At the multivariate stage, three nested logistic regression models were fitted to identify factors that independently influence pregnancy-related supportive behaviors among men. Thus, Model 1, considered the combined effects of only sociodemographic correlates, Model 2 further adjusted for the contribution of sociodemographic variables, while Model 3 assessed the net effects of each covariates by specifying all sociodemographic, socioeconomic and sociocultural variables. Estimates from the logistic regression models were presented as odds ratios with associated 95% confidence intervals and probability values. Statistical significance was determined using a two-tailed probability value less than 0.05. Preliminary assessment based on Variance Inflation Factor (VIF) statistics indicated no multicollinearity issue among the analytical variables. Models performances were compared using critical model parameters including the Log likelihood and Akaike's Information Criterion. Analyses were done using Stata software version 14.2 (24). Meanwhile, transcripts from the focus group session were translated from local dialect to English language and reviewed by professionals to ensure that contextual expressions, concepts and meanings are retained. The transcripts were organized, coded and developed into coherent themes and sub-themes by the authors using inductive and deductive thematic analyses facilitated with NVivo version 12 (QSR (27).

## Results

### Participants distribution by background characteristics

**Figure 1:**
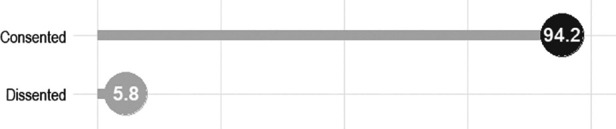
Response rate among 500 total target study population in Modakeke, Southwest Nigeria.

This study targeted 500 potential participants out of which 471 consented to participate and were successfully interviewed translating to 94.2% response rates as depicted in Figure 1. Meanwhile, only 414 respondents with all information relating to pregnancy-related care behavior were included in the present investigation. The distributions of the effective study participants by their background characteristics and status of involvement in pregnancy care are presented in Table 1. As shown in the table, the study included a total of 414 respondents. Most respondents had at least secondary education (51.9% secondary, and 21.5% tertiary) with 8% having no formal education. Most participants were in their thirties (44.2%), married (almost all), and identified as Yoruba. Christians comprised the largest religious group (66%), followed by Muslims (29%) and others (5%). In terms of income, over 25% earned less than 50,000 naira per month, while the majority earned between 50,000 and 100,000 naira. The majority were self-employed (87.4%), with about 40% engaged in manual or other occupations, and 12% were farmers. Concerning antenatal care, over 80% reported their spouses receiving care at hospitals, clinics, or primary healthcare centers, while about 12% mentioned traditional/faith-based facilities. Most respondents (79%) expressed positive views about accompanying their spouse to antenatal care, while 21% had differing opinions. Regarding general pregnancy care involvement, 91% of respondents had positive perceptions, while 9% disagreed or were indifferent to their involvement in pregnancy care.

**Table 1:** Distribution of the study participants by background characteristics in Modakeke, Southwest Nigeria.

	Sample	
	Count	Share
Age group		
20–29	42	10.1
30–39	183	44.2
40–49	140	33.8
50–59	49	11.8
Marital status		
Married	402	97.1
Single dad	12	2.9
Family type		
Polygamous	43	10.4
Monogamous	371	89.6
Ethnicity		
Igbo	11	2.7
Yoruba	394	95.2
Hausa/others	9	2.2
Religion		
Islam	121	29.2
Christianity	271	65.5
Traditional/others	22	5.3
Educational status		
None	31	7.5
Primary	79	19.1
Secondary	215	51.9
Tertiary	89	21.5
Employment status		
Self-employed	362	87.4
Nonself-employed	52	12.6
Occupation group		
Formal	62	15.0
Agricultural	48	11.6
Manual/trading	304	73.4
Income level		
Below 50,000 naira	112	27.1
50,000–100,000 naira	185	44.7
Above 100,000 naira	117	28.3
Place of antenatal care		
Hospital/clinic/PHC	337	81.4
Unskld. birth attendant	50	12.1
Multiple care providers	27	6.5
Attendance of ANC		
Positive perception	328	79.2
Nonpositive perception	86	20.8
Involvement in GPC		
Positive perception	375	90.6
Nonpositive perception	39	9.4

ANC, antenatal care; GPC, general pregnancy care.

### Prevalence of involvement in pregnancy care

**Figure 2:**
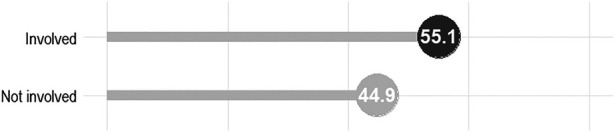
Prevalence of involvement in pregnancy care among men in Modakeke, Southwest Nigeria.

Overall, roughly 55% of the respondents actively supported their spouse during pregnancy period (Figure 2). The results in Table 2 show the involvement rates by specific characteristics. The findings showed the highest and lowest participation rates, respectively, among respondents aged 20–29 (61.9%) and 50–59 (61.9%). More than triple the level of involvement of the single dads was observed among their married counterparts (16.7% vs. 56.2), as those from monogamous families were found to be considerably more involved than those from polygamous families (56.9% vs. 39.5%). Besides, the highest rates were found among men of Igbo ethnic group (72.7%) and those practicing Islam (57.9%). Participation rates increased progressively with level of education with 19.4%, 32.9%, 63.7% and 66.3% corresponding rates among those having no, primary, secondary and tertiary education. Quite similar involvement rates were evident by employment status and occupation group, whereas the level of engagement was found to be more pronounced among men earning less than 50,000 naira monthly relative to others. Meanwhile, place of antenatal care has significant association with men's involvement with the highest level (81.5%) found among those whose spouse had received care from multiple providers. Markedly greater participation rates were observed among respondents who had positive perception about antenatal care attendance and general pregnancy care involvement compared to those who held opposing view. Results from the bivariate logistic regression models presented in the same table corroborate the descriptive findings, indicating significant effects by age group, marital status, family type, educational status, income level, place of antenatal care as well as perception about attendance of antenatal care clinic and involvement in general pregnancy care.

**Table 2:** Estimates of the prevalence and odds of men's involvement in pregnancy care in Modakeke, Southwest Nigeria.

	Involvement prevalence			Involvement odds		
	Est.	Chi-square	*p*-value	UOR	95% CI	*p*-value
Age group						
20–29	61.9	8.177	0.042	1.00	1.00–1.00	Base
30–39	55.7			0.77	0.39–1.54	0.467
40–49	58.6			0.87	0.43–1.77	0.700
50–59	36.7			0.36	0.15–0.84	0.018
Marital status						
Married	56.2	7.367	0.007	1.00	1.00–1.00	Base
Single dad	16.7			0.16	0.03–0.72	0.017
Family type						
Polygamous	39.5	4.682	0.030	0.50	0.26–0.94	0.033
Monogamous	56.9			1.00	1.00–1.00	Base
Ethnicity						
Igbo	72.7	1.807	0.405	2.20	0.57–8.41	0.250
Yoruba	54.8			1.00	1.00–1.00	Base
Hausa/others	44.4			0.66	0.17–2.49	0.539
Religion						
Islam	57.9	2.162	0.339	1.00	1.00–1.00	Base
Christianity	55.0			0.89	0.58–1.37	0.597
Traditional/others	40.9			0.50	0.20–1.27	0.146
Educational status						
None	19.4	42.692	0.000	1.00	1.00–1.00	Base
Primary	32.9			2.04	0.75–5.60	0.164
Secondary	63.7			7.32	2.88–18.61	0.000
Tertiary	66.3			8.19	3.03–22.13	0.000
Employment status						
Self-employed	55.2	0.036	0.849	1.00	1.00–1.00	Base
Nonself-employed	53.8			0.94	0.53–1.69	0.849
Occupation group						
Formal	58.1	0.267	0.875	1.00	1.00–1.00	Base
Agricultural	54.2			0.85	0.40–1.82	0.683
Manual/trading	54.6			0.87	0.50–1.51	0.618
Income level						
Below 50,000 naira	65.2	6.400	0.041	1.00	1.00–1.00	Base
50,000–100,000 naira	51.9			0.58	0.36–0.94	0.026
Above 100,000 naira	50.4			0.54	0.32–0.92	0.025
Place of antenatal care						
Hospital/clinic/PHC	55.2	12.203	0.002	1.00	1.00–1.00	Base
Unskld. birth attendant	40.0			0.54	0.30–0.99	0.047
Multiple care providers	81.5			3.57	1.32–9.66	0.012
Attendance of ANC						
Positive perception	62.5	35.206	0.000	1.00	1.00–1.00	Base
Nonpositive perception	26.7			0.22	0.13–0.37	0.000
Involvement in GPC						
Positive perception	60.0	39.064	0.000	1.00	1.00–1.00	Base
Nonpositive perception	7.7			0.06	0.02–0.18	0.000

Est., estimated prevalence rate; UOR, unadjusted odds ratios; CI, confidence intervals; ANC, antenatal care; GPC, general pregnancy care.

**Table 3:** Logistic regression estimates of the predictors of men's involvement in pregnancy care in Modakeke, Southwest Nigeria.

	Model 1			Model 2			Model 3		
	AOR	95% CI	*p*-value	AOR	95% CI	*p*-value	AOR	95% CI	*p*-value
Age group									
20–29	1.00	1.00–1.00	Base	1.00	1.00–1.00	Base	1.00	1.00–1.00	Base
30–39	0.65	0.31–1.34	0.241	0.69	0.32–1.49	0.345	0.58	0.25–1.31	0.189
40–49	0.79	0.37–1.66	0.529	0.91	0.41–2.02	0.817	0.74	0.31–1.72	0.480
50–59	0.30	0.12–0.74	0.009	0.39	0.15–1.02	0.055	0.36	0.13–1.01	0.053
Marital status									
Married	1.00	1.00–1.00	Base	1.00	1.00–1.00	Base	1.00	1.00–1.00	Base
Single dad	0.12	0.03–0.58	0.008	0.13	0.02–0.73	0.020	0.09	0.02–0.55	0.009
Family type									
Polygamous	0.48	0.25–0.95	0.034	0.64	0.31–1.33	0.229	0.54	0.25–1.18	0.121
Monogamous	1.00	1.00–1.00	Base	1.00	1.00–1.00	Base	1.00	1.00–1.00	Base
Ethnicity									
Igbo	1.91	0.49–7.50	0.353	1.73	0.37–7.98	0.483	1.30	0.25–6.65	0.751
Yoruba	1.00	1.00–1.00	Base	1.00	1.00–1.00	Base	1.00	1.00–1.00	Base
Hausa/others	0.55	0.14–2.27	0.412	1.53	0.31–7.52	0.598	2.74	0.41–18.29	0.299
Religion									
Islam	1.00	1.00–1.00	Base	1.00	1.00–1.00	Base	1.00	1.00–1.00	Base
Christianity	0.86	0.54–1.38	0.530	0.81	0.49–1.35	0.413	0.85	0.50–1.45	0.553
Traditional/others	0.57	0.22–1.50	0.256	0.52	0.18–1.47	0.217	0.67	0.22–2.04	0.476
Educational status									
None				1.00	1.00–1.00	Base	1.00	1.00–1.00	Base
Primary				1.65	0.56–4.89	0.363	1.23	0.37–4.03	0.738
Secondary				6.26	2.25–17.41	0.000	3.55	1.14–10.99	0.028
Tertiary				7.27	2.40–22.01	0.000	4.62	1.37–15.59	0.014
Employment status									
Self-employed				1.00	1.00–1.00	Base	1.00	1.00–1.00	Base
Nonself-employed				0.54	0.23–1.26	0.154	0.47	0.19–1.17	0.103
Occupation group									
Formal				1.00	1.00–1.00	Base	1.00	1.00–1.00	Base
Agricultural				0.86	0.30–2.46	0.784	0.71	0.23–2.17	0.549
Manual/trading				0.61	0.27–1.40	0.242	0.53	0.22–1.30	0.168
Income level									
Below 50,000 naira				1.00	1.00–1.00	Base	1.00	1.00–1.00	Base
50,000–100,000 naira				0.67	0.39–1.15	0.148	0.75	0.43–1.32	0.318
Above 100,000 naira				0.52	0.28–0.95	0.034	0.61	0.32–1.16	0.131
Place of antenatal care									
Hospital/clinic/PHC				1.00	1.00–1.00	Base	1.00	1.00–1.00	Base
Unskld. birth attendant				0.69	0.35–1.37	0.288	0.87	0.42–1.82	0.718
Multiple care providers				3.38	1.15–9.96	0.027	4.97	1.51–16.30	0.008
Attendance of ANC									
Positive perception							1.00	1.00–1.00	Base
Nonpositive perception							0.21	0.12–0.40	0.000
Involvement in GPC									
Positive perception							1.00	1.00–1.00	Base
Nonpositive perception							0.14	0.04–0.51	0.003
LR chi2	25.82			77.66			122.7		
Pseudo R2	0.045			0.136			0.215		
Prob > chi2	0.002			0.000			0.000		
Log likelihood	−271.9			−246.0			−223.5		
Akaike's info. criterion	563.8			532.0			491.0		
Number of respondents	414.0			414.0			414.0		

AOR, adjusted odds ratios; CI, confidence intervals; ANC, antenatal care; GPC, general pregnancy care.

### Predictors of men’s involvement in pregnancy care

#### Quantitative findings

Table 3 presents results from nested logistic regression models analyzing factors predicting pregnancy care involvement in the study population. Emphases are, however, placed on the results from Model 3 of the table that presents the net effect of each predictor. In summary, findings from the model revealed statistically significant variations in the odds of pregnancy care involvement among the male respondents only with respect to marital status, educational status, place of spouse's antenatal care, perception about attendance of antenatal care clinic and perception about involvement in general pregnancy care. According to the results, older men aged 30 and above were marginally less likely to participate in pregnancy care compared to younger ones, with men aged 50–59 being 64% less likely to offer support than those aged 20–29. Single fathers exhibited 91% significantly lower odds of involvement relative to their married counterparts, while men in polygamous families were 0.55 times as likely to be involved as those in monogamous families (*p* > 0.05). Ethnicity and religion had notable but insignificant effects: respondents of Igbo and Hausa/Other ethnicities respectively had 32% and 182% elevated odds of involvement compared to the Yoruba participants, while those practicing Christianity and Traditional/Others religions correspondingly had 15% and 33% reduced odds of participation relative to those practicing Islam. Education had a consistently significant effect on involvement with those possessing secondary and tertiary education exhibiting 255% and 362% greater likelihoods of participation than their uneducated peers. Moreover, the nonself-employed were 0.47 times as likely as the self-employed to provide support during pregnancy (*p* > 0.05), while 29% and 47% lower odds of involvement were associated with agricultural and manual/other occupation, respectively, compared to formal occupation (*p* > 0.05). Also, higher income levels were associated with markedly lower but insignificant likelihoods of being involved. Spousal contact with multiple antenatal care providers predicted 397% increased likelihood of participation than contact with health facility only (*p* < 0.01). Compared to holding positive perception, non-positive perception about accompanying spouse to antenatal care clinic and being involved in general pregnancy respectively correlated with 79% and 86% significantly reduced odds active involvement in pregnancy care.

#### Qualitative findings

This section highlights further findings elicited from focus group discussion focusing on the socioeconomic and sociocultural factors influencing men's involvement in pregnancy-related care in the study setting. The session had in attendance 10 participants out of the 15 that had been invited resulting in 66.6% response rate. Although the discussants differ by age which ranged from 25 to 50 years, 9 identified as Yoruba while one was Igbo. Additionally, 9 participants were from monogamous families, and 3 practiced Islam, with 7 practicing Christianity. Besides, 1 discussant each had no formal education and primary education, 5 had secondary education while the rest had tertiary education. Informal occupation was common among most of the participants with artisans constituting 5, business-men 2, professionals 2, while 1 identified a farmer. The subsequent thematic insights illuminated their attitudes and perceptions towards involvement in pregnancy care, the forms of support they offered during pregnancy and societal perception of men that support their spouse during pregnancy.

### Attitudes and perception towards involvement in pregnancy care

The conversation explored participants attitudes and perspectives in relation to active involvement in pregnancy care and its potential implications. Discussants overwhelmingly expressed positive sentiment towards being active participants in supporting their spouse during pregnancy, emphasizing its significance in ensuring positive perinatal outcomes. They underscored the importance of men providing love, care, and support to their pregnant spouses for both physical and emotional well-being. Additionally, they articulated the crucial role of men in facilitating a peaceful environment and maintaining a strong marital bond.

*“It is very crucial for men to be involved because if the woman feel neglected, it will affect her negatively. Women need spousal support while pregnant; and it is very important to show them all the love and support during the period more than before. Although we should always be concerned about our wives’ wellbeing, our involvement is more important when our wives are pregnant because we are both involved.”* [Civil servant, 47 years]

*“It is very important for men to get involved because it helps a lot especially during child delivery; women need a lot of care from men.”* [Artisan, 32 years]

*“[Men's participation] is very important because it contributes to both the couple's peace of mind, especially someone like me that my job doesn't permit me to be around, my wife needs to feel my presence for that little time. I also provided whatever she demanded during that period.”* [Businessman, 32 years]

*“Men's involvement is very important in many ways, because this process involved both the mother and the baby and there should be a limit to what a woman involved herself because of her health and that of the baby.”* [Farmer, 35 years]

*“It is very important for man to take care of his pregnant woman, if you take care of your woman during her pregnancy period, your love will grow stronger.”* [Artisan, 33 years]

### Types of supports offered spouse during pregnancy

The discussion explored the diverse forms of support offered by participants during their partners’ pregnancies. Participants reflected on the nature and extent of support they provided, and their submissions collectively indicate a spectrum of involvement which encompassed conveyance and accompaniment to antenatal care sessions, performing domestic chores, offering emotional support and assuming responsibility for financial commitments even during challenging periods when physical presence may be constrained by work commitments.

*“It is a good thing to support our wives during pregnancy because we are each other's helper. I love to help her when she is pregnant. I took her to hospital regularly to know her condition and that of the baby.”* [Artisan, 32 years]

*“I assist my wife regularly, especially in fetching water and performing house chores in general, because I know her condition and possible outcome. I know her favorite food and I cooked it for her. Also, I stood by her during childbirth.”* [Businessman, 45 years]

*“I assisted my wife in some ways. For instance, I took care of our first child during the pregnancy period of our second child. I cooked and did house chores. I took care of the home too unless I am not around.”* [civil servant, 40 years]

*“I supported my wife in reminding her the date for antenatal care, calling her at the time set for her to take medication, and render other little helps I can anytime I am around because my job doesn't permit me to be with her at all times.”* [Businessman, 34 years]

### Societal perception of men that support their spouse during pregnancy

Conversation about societal opinions about men who assist their spouses during pregnancy uncovered a range of negative perceptions, stereotypes and stigmatization. The discussants identified traditional gender roles, cultural norms, and jealousy as contributors to the hostile views of men rendering support during pregnancy, particularly in the context of accompanying their spouses to antenatal care visits. Participants unanimously echoed societal biases, portraying men in supportive roles as jobless, lacking focus or manipulated, thus shedding light on the multifaceted challenges faced by men engaging in pregnancy-related care.

*“Many people said my wife has manipulated me. People perceived men that accompany their pregnant spouse to antenatal visit as jobless or lacking focus.”* [Civil servant, 40 years]

*“Most people see men that support their wives as different from the rest of them. They tag men that accompany their wives to antenatal care visit as jobless.”* [Artisan, 50 years]

*“Many of these people perceived men that support their spouse have been manipulated for rendering the support to their pregnant wives. People have said it to my face that I am jobless for accompanying my spouse to antenatal visit.”* [Artisan, 32 years]

“It is a crime for a man to provide domestic support to his wives in my own area. If a man did such, they would make jest of him. People perceived men that escort their pregnant spouse to antenatal visit as dunce and jobless.” [Artisan, 26 years]

*“Many of those people made jest of me while I rendered support for my spouse; especially, domestic ones. In my area, people perceive men that attend antenatal care visits with their pregnant wives as a jobless man.”* [Farmer, 35 years]

*“My own opinion is indifferent about people's view of men that support their wives during pregnancy period.”* [Artisan, 33 years; Businessman, 34 years; Civil servant, 47 years]

## Discussion

This study examined the level and correlates of men's involvement in pregnancy care in Modakeke, Southwest Nigeria. The study revealed a moderate participation rate in pregnancy-related care among the study respondents with roughly 55% found to have accompanied their spouse to antenatal care clinic and offered a combination of emotional, financial, and domestic supports. This finding is consistent with reports from similar social contexts of Agege, Southwest Nigeria (7) and Ibadan, Southwest Nigeria (11), which correspondingly indicated approximately 59% and 57% men's involvement rates but at variance with evidence from Benin City, Southsouth Nigeria (8), Dodoma Region, Central Tanzania (9), the Bench Sheko zone, Southwest Ethiopia (10), Kashan city, Iran (13), Sekondi, Ghana (14), and Kano, Northern Nigeria (25) which respectively documented roughly 27%, 20%, 38%, 62%, 70% and 72% levels of participation in maternity care. Differences in conceptual definition of male partner's involvement, healthcare systems and sociocultural contexts may have accounted for the observed variations across studies. Moreover, the level of involvement varied markedly by participants’ background characteristics as typically obtained in previous works (10, 18, 22, 25).

The level of involvement in pregnancy care found in this study fell short of expectation notwithstanding its positive correlation with improved perinatal outcomes as documented in the literature (3, 6, 28). Researchers have argued that involving husbands/partners and communities in antenatal care services in a health facility and community settings can enhance improved maternal and child health outcomes (29). For instance, a study by Alemi et al. (3) established that accompanying a pregnant woman to antenatal care raised the odds of commencing antenatal visits during the first trimester, adequate use of antenatal care services, health facility delivery and presenting for postnatal check-ups. Reports from numerous studies similarly indicated that male participation in pregnancy-related care predicted increased likelihoods of maternal health service utilization, continuum of care completion, infectious diseases prevention, obstetric complications awareness, birth preparedness involvement, reduction in postpartum depression and postpartum modern contraception utilization (5, 6, 26, 28, 30–32). Thus, active male partner's support during pregnancy can enhance the quality of life of mother and child and facilitate reduction in the rates of maternal and child morbidity and mortality in low- and middle-income countries.

The study found a substantial yet statistically insignificant age difference in men's participation in pregnancy care with younger respondents exhibiting greater involvement compared to their older counterparts. This finding aligns with outcomes reported in investigations done in Ife Central, Southwest Nigeria (33), Benin-City, Southsouth Nigeria (8), Ungogo, Northern Nigeria (23), and Asmara, Eritrea (22) but contradicts evidence from studies conducted in Bench Sheko zone, Southwest Ethiopia (10) where being older was associated increased tendency of being involved in maternity care. The observed age disparity may be ascribed to distinctions in socioeconomic attributes among the age cohorts. For instance, higher educational attainment among younger men likely underlies their greater engagement in pregnancy care. The finding could also be attributed to differences in generational perspectives, with older men potentially adhering to sociocultural norms that assign primary caregiving roles to women.

Moreover, findings from the study revealed significant differential in men's engagement in pregnancy care by marital status, whereas marginal but striking disparity was evident with regards to family structure. Generally, married men and those within monogamous family arrangements demonstrated elevated levels of involvement in contrast to single fathers and those from polygamous family setups. Similar findings have been widely documented in the past studies where stable and monogamous unions have been linked with increased level of male partner's involvement (14, 23, 33, 34). Men in stable and monogamous unions may experience a more conducive environment for shared responsibilities leading to greater participation in pregnancy care. Also, societal expectations and cultural norms surrounding familial roles may influence the level of engagement with being married and monogamous family structure aligning more closely with prevailing expectations. Moreover, the financial and emotional support systems inherent in stable and monogamous family settings could contribute to higher levels of involvement in pregnancy care activities.

Ethnicity and religion played notable but insignificant roles in differentiating the likelihoods of being supportive to pregnant spouse. Participants of Igbo and Hausa/Other ethnic background tend to be more involved in supporting their wives during pregnancy compared to their Yoruba counterparts. Also, those practicing Christianity and Traditional/Others religions exhibited lower odds of active engagement with their spouse in relation to those practicing Islam. These agree with findings from other studies from Nigeria (11, 23, 25) and elsewhere (10, 22). For instance, investigation by Falade-Fatila and Adebayo (11) in Ibadan, Southwest Nigeria indicated that men who identified as Christians and Igbo, Hausa and Edo tribes were more involved in pregnancy-related care than the Muslims and native Yoruba men. The greater involvement observed among the nonindigenous tribes’ men may be attributed to the nuclear structure of most immigrant families that typically necessitates active participation of men in caregiving activities as other primary caregivers available to their indigenous counterparts are often absent.

Findings from the study revealed a positive influence of education on level of involvement. Notably, higher levels of education correspond to increased involvement, with men possessing secondary and tertiary education exhibiting significantly greater participation in pregnancy care in relation to their counterparts with no formal or primary education in consonance with studies done in diverse contexts (7, 8, 10–13, 22, 23). Education is the most consistent social determinant health of a society as it informs knowledge, attitude and practice that promotes positive health behaviors and outcomes (35). This is evidenced by numerous studies addressing various public health issues including pregnancy-related care (7, 23, 25, 30, 33, 35, 36). Education substitutes traditional gender norms with liberal attitudes of shared responsibility, provides access to resources and equips individuals with relevant knowledge across various life domains, including reproductive health, thus, fostering increased male partner's engagement during pregnancy (12, 37).

Although our analyses indicate employment status, occupation group, and income level are not robust predictors of men's involvement in pregnancy care in the study population, striking disparities were discernible across the socioeconomic strata. The results suggest that paid employment, informal occupation, and higher income levels undermines men's propensity to participate in pregnancy-related care, corroborating previous findings (7, 14, 16, 38, 39). The predominance of higher education among the study participants could account for the trivial explanatory power of employment, occupation and income covariates. Roughly three-fourth (73%) of the study population had secondary education and this group constituted the majority across the categories of occupation (63%–79%) and income (67%–80%), for instance. Nevertheless, the reduced odds of involvement in pregnancy care observed among non-self-employed men may have stemmed from work-related constraints and inflexible schedules associated with paid employment. Likewise, high-income individuals are more likely to occupy positions demanding extensive time dedication and might face challenges in allocating time to caregiving roles. Such constraints may impede their ability to actively engage in pregnancy care activities, attend antenatal visits, or provide necessary support to their spouses as was echoed during focus group discussion and reported in extant works (20).

Findings pertaining to involvement in pregnancy care as influenced by the place of antenatal care revealed compelling insights. Participants whose spouses received care from unskilled practitioners had marginally lower odds of being involved, whereas those whose partners had contacts with multiple care providers exhibited nearly four-fold tendency of being involved in relation to their peers whose spouse received care from health facilities. The reduced involvement associated with unskilled service providers may stem from perceived inadequacies in healthcare quality and the traditional association of these attendants with less formalized, potentially less inclusive care. Conversely, the heightened involvement with multiple care providers may indicate that diversified, comprehensive care fosters a more inclusive approach, fostering men's active participation in the pregnancy care process. This evidence constitute a significant addition to literature on factors determining male partner's participation in obstetric care, underscoring the need for further research to uncover rationales underlying the observed relationship.

Furthermore, our investigation underscores the pivotal role of individual perceptions in shaping engagement in pregnancy care. Results from the bivariate and multivariate analyses consistently indicate that a positive perception regarding the act of accompanying a spouse to antenatal care and providing general pregnancy-related care have significant positive influence on men's actual involvement in pregnancy care. A few research has shown that men with positive attitudes towards supportive behaviors are more inclined to participate in pregnancy care activities (13, 19, 22, 33). Positive male perceptions may correlate with a greater sense of responsibility and commitment towards the well-being of the spouse and unborn child. Also, it may signify an alignment with progressive societal values that recognize and encourage men's active roles in reproductive health, thereby fostering a culture of inclusive and collaborative pregnancy care practices. Although this study found that most of the study participants expressed positive attitudes towards supportive behaviors, perceived challenges to active involvement included traditional gender norms, social stigma, ridicule, unconducive health facility environment and exclusion of men from maternity care activities in agreement with evidence from earlier investigations (5, 11, 20, 31, 33, 34, 39–42).

## Conclusions and recommendations

This investigation underscores the complex interplay of sociodemographic and sociocultural factors in shaping men's involvement in pregnancy care. The study revealed marital instability, lack of adequate formal education, use of single care provider during antenatal period as factors significantly impeding male participation in the study area. Also, positive perceptions about attending antenatal care clinic with spouse and providing general pregnancy-related care emerged as critical predictors of active involvement of male partner. Nevertheless, substantial but statistically insignificant disparities in level of engagement were evident in relation to family type, religion, ethnicity, employment status, occupational group and level of income. These findings portend critical directions for community-based interventions aimed at improving male partner participation in pregnancy-related care in the study setting. There is the need to develop targeted interventions and implement support programs specifically designed to encourage and facilitate involvement in pregnancy care among older men and single fathers. Such interventions and programs should provide resources, information and community networks to help these groups navigate their roles during pregnancy. Also, it is imperative to launch educational campaigns targeting men with lower educational attainment, emphasizing the benefits of active involvement in pregnancy care. These campaigns should empower them with the knowledge and skills necessary to support their partners during pregnancy and highlight the positive impacts of such pregnancy-related support. Furthermore, policymakers and community-based healthcare providers should implement awareness-raising campaign challenging norms hindering men's participation and disseminating positive narratives about the cross-cutting benefits of men's supportive roles during pregnancy. Implementing these recommended initiatives has the potential to promote safe motherhood and enhance maternal and child well-being and quality of life.

### Strengths and limitations

To the best of our knowledge, this study is the first to explore partner involvement in pregnancy care in study area and one of the few studies to have examined the subject in Nigeria in contemporary times. The selection criteria were designed to capture most recent experience of the study participants and reduce recall bias, while the mixed method approach employed enriched the study. However, the cross-sectional design of the study constrained causal inferences, thereby limiting our findings as indicative of association between the outcome and selected correlates. Besides, the responses are self-reported which may engender social desirability bias, particularly regarding involvement. Furthermore, resource constraints limited the qualitative aspect of the study to one focus group discussion session, possibly compromising the generalizability and saturation of evidence. Thus, by conducting multiple focus group discussions and employing other qualitative methods such as in-depth interview and key informant interview, future studies can offer more comprehensive insights into the perspectives and lived experiences of male partners with respect to providing pregnancy-related supports in the study setting.

## Data Availability

The datasets presented in this article are not readily available due to future use of the datasets. Requests to access the datasets should be directed to elhakim.ibrahim@yahoo.com.
